# Adaptation of the 5-choice serial reaction time task to measure engagement and motivation for alcohol in mice

**DOI:** 10.3389/fnbeh.2022.968359

**Published:** 2022-09-16

**Authors:** Phillip Starski, Danielle Maulucci, Hunter Mead, Frederic Hopf

**Affiliations:** ^1^Department of Psychiatry, Indiana University School of Medicine, Indianapolis, IN, United States; ^2^Department of Psychology, Indiana University-Purdue University Indianapolis (IUPUI), Indianapolis, IN, United States

**Keywords:** alcohol, 5-choice serial reaction time task, alcohol preference, intermittent alcohol access, behavioral engagement

## Abstract

Alcohol use disorder (AUD) is related to excessive binge alcohol consumption, and there is considerable interest in associated factors that promote intake. AUD has many behavioral facets that enhance inflexibility toward alcohol consumption, including impulsivity, motivation, and attention. Thus, it is important to understand how these factors might promote responding for alcohol and can change after protracted alcohol intake. Previous studies have explored such behavioral factors using responding for sugar in the 5-Choice Serial Reaction Time Task (5-CSRTT), which allows careful separation of impulsivity, attention, and motivation. Importantly, our studies uniquely focus on using alcohol as the reward throughout training and testing sessions, which is critical for beginning to answer central questions relating to behavioral engagement for alcohol. Alcohol preference and consumption in male C57BL/6 mice were determined from the first 9 sessions of 2-h alcohol drinking which were interspersed among 5-CSRTT training. Interestingly, alcohol preference but not consumption level significantly predicted 5-CSRTT responding for alcohol. In contrast, responding for strawberry milk was not related to alcohol preference. Moreover, high-preference (HP) mice made more correct alcohol-directed responses than low-preference (LP) during the first half of each session and had more longer reward latencies in the second half, with no differences when performing for strawberry milk, suggesting that HP motivation for alcohol may reflect “front-loading.” Mice were then exposed to an Intermittent Access to alcohol paradigm and retested in 5-CSRTT. While both HP and LP mice increased 5-CSRTT responding for alcohol, but not strawberry milk, LP performance rose to HP levels, with a greater change in correct and premature responding in LP versus HP. Overall, this study provides three significant findings: (1) alcohol was a suitable reward in the 5-CSRTT, allowing dissection of impulsivity, attention, and motivation in relation to alcohol drinking, (2) alcohol preference was a more sensitive indicator of mouse 5-CSRTT performance than consumption, and (3) intermittent alcohol drinking promoted behavioral engagement with alcohol, especially for individuals with less initial engagement.

## Introduction

Excessive alcohol consumption is a prevalent activity that may progress to Alcohol Use Disorder (AUD), and ∼3/4th the ∼$250 billion/year cost of drinking in the US comes from the ∼1/7th of adults who binge (CDC, 2014). Excessive intake can contribute strongly to the substantial harms of alcohol, including enhanced risk of drinking problems (Esser et al., 2014; Grant et al., 2015; Gowin et al., 2017), while reducing excess intake lowers health risks and relapse (Dawson et al., 2005; Moos and Moos, 2006; Rehm et al., 2009). Higher risk for binge drinking has been linked to high trait impulsivity (Poulos et al., 1995; Crews and Boettiger, 2009; Schumann et al., 2011), and non-dependent drinkers with higher self-reported impulsive behavior achieve higher blood alcohol levels during free-access self-administration, and experience greater euphoria from alcohol (Vaughan et al., 2019). Impulsivity is complex construct (Meda et al., 2009; MacKillop et al., 2016; Strickland and Johnson, 2021), with variants related to motor (impulsive action) and cognitive (impulsive choice) functions (see section “Discussion”), and is considered an important risk factor for AUD. As this disorder develops, it is accompanied by significant changes in cognitive behavioral control (Tapert et al., 2004; Wilcox C. E. et al., 2014). The desire for intoxication and the increased tolerance of adverse consequences are examples of motivational changes in people with AUD (Larimer et al., 1999; Lau-Barraco et al., 2017; Radke et al., 2021). Further, an “attentional bias” will typically develop that promotes behavior toward alcohol cues over natural rewards (Fadardi and Cox, 2006; Monem and Fillmore, 2019; Heitmann et al., 2020). Together, impulsivity, motivation, and attention are key aspects of behavioral engagement with alcohol that we seek to investigate, especially changes in such responding after chronic alcohol use.

The 5-choice serial reaction time task (5-CSRTT) is a multifaceted behavioral paradigm that has been thoroughly characterized in rodents to elucidate impulsive, attentional, motivational, and perseverative behavior in the same session (Robbins, 2002; Bari et al., 2008; Semenova, 2012). Thus, the 5-CSRTT is valuable for assessing a broad range of measures of behavioral performance, when compared to many other tasks. Interestingly, a 5-CSRTT version adapted for humans predicts higher alcohol intake in more impulsive individuals, suggesting high translational value (Sanchez-Roige et al., 2014a). While determining clear correlations between behavioral factors in human studies remains challenging, rodent studies give the ability to dissect important contributors to behavioral engagement for alcohol. However, to date, studies examining the relation of alcohol and impulsivity have primarily determined 5-CSRTT responding for sugar in relation to alcohol exposure (Semenova, 2012; Sanchez-Roige et al., 2014a,b; Irimia et al., 2015; Pena-Oliver et al., 2015; Starski et al., 2019, 2020).

Here, we have uniquely adopted the 5-CSRTT to have alcohol as the reward, allowing us greater precision in identifying the nature of behavioral engagement, with the goal of understanding how impulsivity, motivation, and attention for alcohol might relate to preference or consumption. Interestingly, we found that 5-CSRTT performance was significantly related to alcohol preference rather than consumption level. Thus, it is interesting that, in addition to high trait impulsivity, people at risk for binging have higher alcohol preference (Poulos et al., 1995; Crews and Boettiger, 2009; Schumann et al., 2011), and impulsivity can be linked to preferences in rodents (Oberlin and Grahame, 2009; Dick et al., 2010; Lejuez et al., 2010; Adams et al., 2013; Elder et al., 2019; Herman and Duka, 2019) (see section “Discussion”). In addition, after intermittent alcohol consumption, mice overall increased their performance, but this was especially pronounced in initially low-responding mice, suggesting that protracted drinking may be particularly hazardous for individuals with lower initial drive for alcohol. Finally, we also performed several days of 5-CSRTT responding for strawberry milk, with our previously used methods (Starski et al., 2019, 2020). Sweet milk responding was higher than alcohol and with greater accuracy, did not relate to alcohol preference, and had minimal changes with intermittent drinking, suggesting important specificity in the alcohol-engagement relationship. We provide herein a robust and valuable model to help understand inter-relationships between different aspects of engagement for alcohol, and how they could be altered by intermittent drinking, which together promote excessive intake.

## Materials and methods

### Animals

Forty-eight male C57BL/6J mice from Jackson Laboratories Inc., were individually housed, starting at 8 weeks old, in standard Plexiglass cages with *ad libitum* access to food and water until water restriction. Mice were maintained in a 12 h:12 h reverse light-dark cycle. Animal care and handling procedures were approved by the Indiana University Institutional Animal Care and Use Committee in accordance with NIH guidelines.

#### 5-Choice serial reaction time task

**FIGURE 1:**
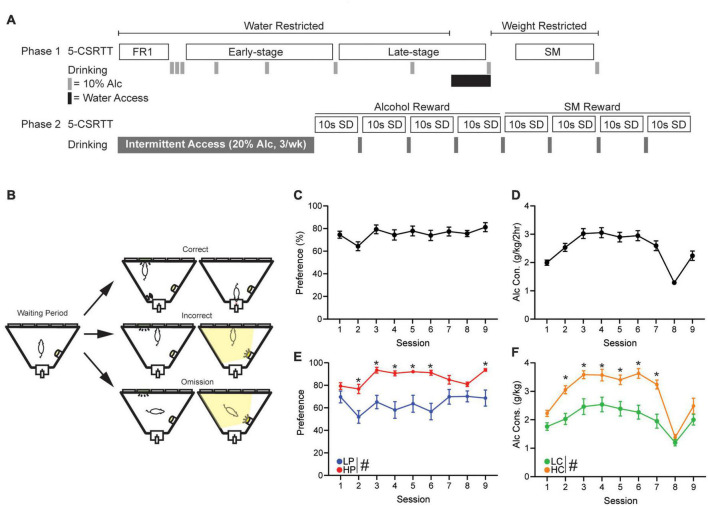
5-Choice Serial Reaction Time Task training schedule and alcohol drinking. **(A)** Schedule of 5-CSRTT and drinking behavior. **(B)** Graphic detailing a correct, incorrect, and omission within the 5-CSRTT. Briefly, a head entrance into an illuminated reward tray initiates the waiting period. When the waiting period ends, a light will appear in 1 of 5 ports. A touch in the illuminated port is a correct response and will result in reward delivery. A touch in an unlit port is an incorrect response and will cause a punishing flash of light and no reward. If the lit port extinguishes and the limited hold period elapses, this will result in an omission causing a punishing flash and no reward. Overall **(C)** alcohol preference and **(D)** alcohol consumption of all mice, across the 9 days of 2-h DID two-bottle choice drinking (gray bars). **(E)** Alcohol preference when mice were separated by median split of preference (HPvLP: *F*_1,46_ = 82.06, *p* < 0.0001). *Post hoc* revealed significance on sessions 2–6 and 9. **(F)** Alcohol consumption when mice were separated by median split of consumption (*F*_1,46_ = 81.31, *p* < 0.0001; time: *F*_6_._11,279_._4_ = 18.30, *p* < 0.0001; interaction: *F*_8,366_ = 2.22, *p* = 0.0253). *Post hoc* revealed significance on sessions 2–7. Session 8 reflects the time in which the mice were given 1 week of water access before being water restricted again. ^#^*p* < 0.05 for group effect. *p < 0.05 *post hoc* significance. All data are expressed as ± standard error mean.

**(A)** A detailed description of early-stage, late-stage, and strawberry milk (SM) training can be found in Supplementary methods. All mice were trained and tested under a 10 s stimulus duration (SD) and 5 s intertrial interval (ITI): this was done to reduce challenge within the task, since this is, to our knowledge, the first investigation using an intoxicant (10% alcohol) as the reward in 5-CSRTT. Figure 1A shows the overall timeline of studies, and Figure 1B and Supplementary Figure 1 give visual representations of several typical session events.

**(B)** 5-Choice serial reaction time task motivators: For alcohol sessions, mice were water restricted because we wanted to heighten their response levels, and, importantly, in the oral modality that is used for alcohol; in other words, water restriction was more ethological as comparison for alcohol versus food restriction. For SM sessions, SM is much more of a nutrient, while alcohol has calories it is consumed more as an intoxicant. For further information see Supplementary discussion.

**(C)** Post-Intermittent Access Testing (Figure 1A, Phase 2). For these studies, mice drank IA2BC interspersed with 5-CSRTT testing (see Supplementary Figure 9). Mice were given four sessions for alcohol reward and 4 for a SM reward using a 10 s SD and 5 s ITI duration. Mice continued intermittent access alcohol intake across the testing weeks, resulting in approximately 6 weeks of total intermittent access, and were not weight restricted or continuously water restricted. Importantly, 5-CSRTT testing occurs after a IA2BC session. Briefly, a testing day begins with removal of the 24 h 20% alcohol bottle and no water access until, 5–9 h later, we begin behavioral testing; this timing ensures mice were performing during acute withdrawal (Hwa et al., 2011; Metten et al., 2018). Water bottles were given immediately after behavioral testing was completed for the day and remained until the next IA2BC session (Supplementary Figure 9). For analysis we excluded the first day to remove potential burst in behavior from reintroducing the mice to the task.

### Drinking in the dark and intermittent access

**(A)** Throughout 5-CSRTT training, mice were given weekend drinking in the dark (DID) session of 10% alcohol for 2 h to promote response to the reward (Figure 1A, Phase 1). Custom-built, low drip sipper tubes were used to reduce dripping from overactive mice that may climb on the cage. These tubes consisted of a Falcon 15 mL conical tube (Fisher Scientific, Hampton, NH, USA) with the bottom cone cut and filed down. A sipper (Ancare Corp., Bellmore, NY, USA) was then placed inside the tube and shrink-wrapped using a heat gun. A rubber stopper (size:0#, StonyLab, Nesconset, NY, USA) was used to plug the opposite end. Bottles were weighed before and after sessions and consumption was calculated using the weekly weight of the mouse. Preference was calculated as the total amount of alcohol consumed divided by total liquid consumed.

**(B)** Similar to Lei et al. (2019), mice were given 24 h access to 20% alcohol every Sunday, Tuesday, and Thursday starting at 7:00 a.m. (Figure 1A, Phase 2). Consumption was calculated using the weekly weight of the mouse. During non-alcohol days, two-bottles filled with water were present to maintain familiarity with the bottles.

### Statistical analysis

The 48 mice were categorized as High/Low preference or consumption after the Late-Stage sessions and once the DID behavior was completed. Specifically, alcohol consumption and preference were calculated for each animal based on the overall average of the nine DID sessions. For classifying preference groups, mice were ordered from greatest preference to least preference and a median split was used to divide the 48 mice into two equal-sized groups. For classifying consumption groups, the same mice were instead ordered from greatest consumption to least consumption and a median split was used to divide mice into two equal-sized groups. The same sets of analyses were conducted for preference and consumption. 5-CSRTT and alcohol consumption studies were analyzed by two-way repeated measures analysis of variance (ANOVA) followed by Bonferroni’s multiple comparisons test where appropriate. Sphericity was not assumed, and the Geisser–Greenhouse correction was used. For missing data points (spill during drinking, or animal non-responding), a mixed-model analysis of variance was used. All group analyses of pre-IA2BC versus post-IA2BC and performance changes were tested for normal distribution (Shapiro–Wilk test), and then an appropriate test (parametric or non-parametric) was used to measure differences (*t*-test, Mann–Whitney, Paired *t*-test, Wilcoxon). Non-parametric data is reported simply in the main text, with specific values in the Supplementary material. All statistical analyses were calculated using Prism 9.0 software (Graphpad Software Inc., San Diego, CA, USA), with significance set at *p* < 0.05.

## Results

### Mice with high alcohol preference or consumption show higher engagement in early-stage training

To better understand the relationship between alcohol drinking behavior and 5-CSRTT performance, drinking preference and consumption was calculated from the first nine 2 h-DID two-bottle-choice sessions (Figures 1C,D). A median split was then performed to compare response patterns in High alcohol Preference (HP, *n* = 24) versus Low Preference (LP) mice (*n* = 24, Figure 1E), and a separate median split was performed to compare high consumption (HC, *n* = 24) versus low consumption (LC, *n* = 24) mice (Figure 1F). For all subsequent analyses, performance measures were analyzed separately by preference and by consumption.

**FIGURE 2:**
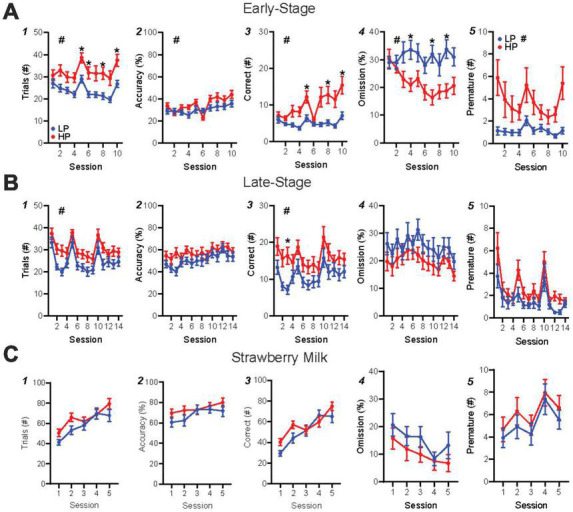
HP versus LP early-stage and late-stage alcohol, and SM training in the 5-CSRTT. **(A)** Early-stage performance showed differences in trials, accuracy, correct (*post hoc* significance on session 3), % omission, and premature between HP and LP mice. **(B)** Late-stage performance displayed HP-LP differences in trials and correct, but not accuracy, % omission or premature. **(C)** SM performance showed no differences in any metric. *n* = 24/group for preference. ^#^*p* < 0.05 for group effect. *p < 0.05 *post hoc* significance. All data are expressed as ± standard error mean.

Each 5-CSRTT session involved unlimited trials in an hour period, where a nosepoke to an illuminated stimulus in one of five ports led to rear reward delivery (detailed further in Figure 1B legend). During the first 10 days of training (“early-stage”), HP mice performed significantly more trials than LP mice (Figure 2A1, HPvLP: *F*_1,46_ = 11.36, *p* = 0.0015; time: *F*_1_._93,88_._74_ = 9.59, *p* = 0.0002), with higher accuracy (Figure 2A2, HPvLP: *F*_1,46_ = 4.84, *p* = 0.0329; time: *F*_4_._81,221_._2_ = 6.57, *p* < 0.0001; interaction: *F*_9,414_ = 1.96, *p* = 0.0421) and significantly more correct responses compared to LP mice (Figure 2A3, HPvLP: *F*_1,46_ = 11.16, *p* = 0.0017, time: *F*_1_._76,80_._8_ = 8.5, *p* = 0.0008; interaction: *F*_9,414_ = 4.57, *p* < 0.0001). In addition, the percent of trials that were omissions in early-stage was lower in HP mice (Figure 2A4, HPvLP: *F*_1,46_ = 11.4, *p* = 0.0015; interaction *F*_9,414_ = 2.48, *p* = 0.0091). However, raw premature responses were significantly higher in HP mice (Figure 2A5, HPvLP: *F*_1,46_ = 6.96, *p* = 0.0113; time: *F*_1_._39,64_._07_ = 4.13, *p* = 0.0334; interaction: *F*_9,414_ = 2.48, *p* = 0.0091), as were the percentage of premature responses (Supplementary Figure 3G, HPvLP: *F*_1,46_ = 7.04, *p* = 0.0109; time: *F*_2_._71,124_._6_ = 2.93, *p* = 0.0411; interaction: *F*_9,414_ = 3.27, *p* = 0.0007). Together, these suggest that HP have greater engagement than LP mice early in training, with greater number of trials, correct, and accuracy, and also fewer omissions and greater premature responding (which could reflect greater impulsivity, or simply greater engagement with the task).

Similar to our HP versus LP comparison, we found that mice with HC overall performed more in early training trials than LC mice (Supplementary Figure 2A1, HCvLC: *F*_1,46_ = 4.12, *p* = 0.0482; time: *F*_1_._81,83_._1_ = 9.56, *p* = 0.0003) and had more correct responses than LC mice (Supplementary Figure 2A3, HCvLC: *F*_1,46_ = 4.57, *p* = 0.0380; time: *F*_1_._67,76_._91_ = 8.11, *p* = 0.0013; interaction: *F*_9,414_ = 2.25, *p* = 0.0185), although with no *post hoc* differences in any session. In addition, and unlike HP versus LP, HC, and LC mice were not different in accuracy (Supplementary Figure 2A2, HCvLC: *F*_1,46_ = 1.34, *p* = 0.2528; time: *F*_4_._55,209_._3_ = 6.47, *p* < 0.0001) or percentage of omissions (Supplementary Figure 2A4, HCvLC: *F*_1,46_ = 3.44, *p* = 0.0699). Together, these suggest that differences in performance early in training were more related to alcohol preference differences rather than consumption.

### Preference predicts performance, while alcohol consumption does not, during late-stage training

In late-stage training, we continued with a 10 s SD and 5 s ITI, since we wanted to explore potential behavioral differences under simpler task requirements in this first-time assessment of 5-CSRTT with alcohol as the reward. HP and LP mice continued to show significant performance differences in late-stage training, while HC and LC mice did not. HP mice performed more trials (Figure 2B1, HPvLP: *F*_1,46_ = 5.93, *p* = 0.0188; time: *F*_7_._74,355_._9_ = 18.91, *p* < 0.0001) and correct responses (Figure 2B3, HPvLP: *F*_1,46_ = 4.84, *p* = 0.0329; time: *F*_7_._2,331_._4_ = 9.11, *p* < 0.0001) than LP mice. However, by late-stage training, there were no differences between preference groups for accuracy (Figure 2B2, HPvLP: *F*_1,46_ = 1.92, *p* = 0.1730; time: *F*_7_._52,345_._8_ = 5.37, *p* < 0.0001), omissions (Figure 2B4, HPvLP: *F*_1,46_ = 1.88, *p* = 0.1772; time: *F*_6_._81,313_._1_ = 3.01, *p* = 0.0049), raw premature responses (Figure 2B5, HPvLP: *F*_1,46_ = 3.801, *p* = 0.0573; time: *F*_4_._90,225_._4_ = 10.48, *p* < 0.0001), or percentage of premature responses (Supplementary Figure 3H, HPvLP: *F*_1,46_ = 3.96, *p* = 0.0525; time: *F*_8_._71,400_._5_ = 5.43, *p* < 0.0001). In contrast, HC and LC mice did not show differences in trials performed (Supplementary Figure 2B1, HCvLC: *F*_1,46_ = 1.71, *p* = 0.1979; time: *F*_7_._8,359_ = 19.10, *p* < 0.0001), accuracy (Supplementary Figure 2B2, HCvLC: *F*_1,46_ = 0.119, *p* = 0.7319; time: *F*_7_._56,347_._8_ = 5.34, *p* < 0.0001), number of correct responses (Supplementary Figure 2B3, HCvLC: *F*_1,46_ = 0.717, *p* = 0.4016; time: *F*_7_._45,342_._9_ = 9.2, *p* < 0.0001; interaction: *F*_13,598_ = 1.89, *p* = 0.0286), or omissions (Supplementary Figure 2B4, HCvLC: *F*_1,46_ = 0.139, *p* = 0.7115; time: *F*_6_._74,309_._9_ = 3.03, *p* = 0.0048). Together, our data suggest, perhaps surprisingly, that preference is a better indicator of established (later-stage) performance under a more basic version of 5-CSRTT than consumption, since HP had more trials and correct responses than LP, while HC and LC were not different.

Estimated intake during sessions was calculated by the 200 μl initial “free reward” and each subsequent correct response that delivers 20 μl. When calculating average intake by preference, there was a trend that HP mice may have higher intake as they get more correct responses compared with LP mice (Supplementary Figure 4N, HPvLP: *F*_1,46_ = 2.842, *p* = 0.0986; time: *F*_2_._644,121_._6_ = 7.669, *p* = 0.0002). When analyzed by consumption, there were no differences in intake levels (Supplementary Figure 4O, HCvLC: *F*_1,46_ = 0.1393, *p* = 0.7107; time: *F*_2_._656,122_._2_ = 8.002, *p* = 0.0001).

### Unlike alcohol, performance for strawberry milk reward is not related to alcohol preference

After late-stage alcohol testing, mice were switched for five sessions to a strawberry milk (SM) reward in the 5-CSRTT to determine whether preference-related performance for alcohol (Figures 2A,B) might be related to more basic differences in motivation for reward learning. Overall, mice had more than twice the number of responses for SM relative to alcohol [Supplementary Figure 4M, paired *t*-test, *t*(94) = 10.38, *p* < 0.0001]. However, there were no differences in any response measure between HP and LP mice, including in number of trials (Figure 2C1, HPvLP: *F*_1,46_ = 1.571, *p* = 0.2163; time; *F*_2_._76,127_._0_ = 42.46, *p* < 0.0001; interaction: *F*_4,184_ = 3.017, *p* = 0.0193), accuracy (Figure 2C2, HPvLP: *F*_1,46_ = 1.278, *p* = 0.2639; time: *F*_2_._81,129_._3_ = 7.62, *p* = 0.0001), correct responses (Figure 2C3, HPvLP: *F*_1,46_ = 1.242, *p* = 0.2709; time *F*_2_._73,125_._5_ = 43.63, *p* < 0.0001), omissions (Figure 2C4, HPvLP: *F*_1,46_ = 1.119, *p* = 0.2956; time: *F*_2_._67,122_._9_ = 7.03, *p* = 0.0013), raw premature responses (Figure 2C5, HPvLP: *F*_1,46_ = 0.7796, *p* = 0.3818; time: *F*_3_._41,157_._0_ = 4.471, *p* = 0.0032), or percentage of premature responses (Supplementary Figure 3I, HPvLP: *F*_1,46_ = 0.6390, *p* = 0.4282). Responding for SM was also unrelated to higher versus lower consumption (Supplementary Figures 2, 3). Importantly, these findings suggest that HP and LP mice had similar ability to learn and perform for a high-value reward, and thus that reduced alcohol responses in LP mice did not reflect differences in basic reward behavior, but, instead, a difference in engagement in responding for alcohol.

### Reward latency is increased during alcohol sessions, but not in strawberry milk, due to occasional longer reward latency trials

**FIGURE 3:**
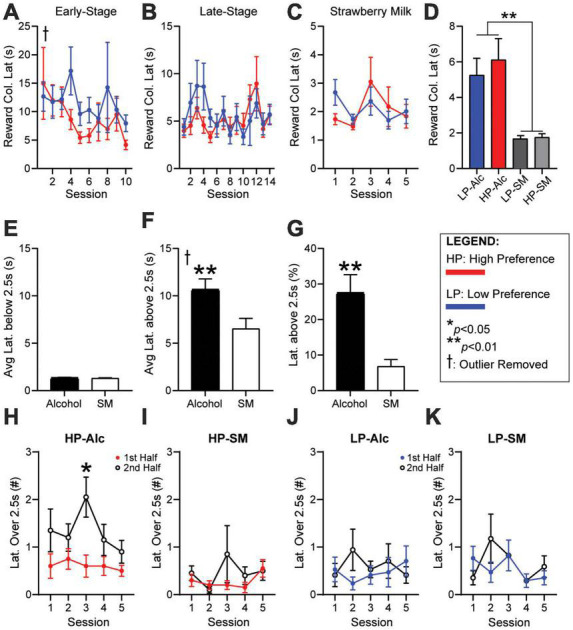
Reward latencies are longer during the second half of HP sessions for alcohol, not SM. No differences in **(A)** early-stage, **(B)** late-stage, or **(C)** SM reward latency. **(D)** Average reward latency was greater for alcohol (HP/LP) compared to SM sessions (HP-SM/LP-SM). **(E)** Average latencies below 2.5 s were similar for alcohol and SM. **(F)** Average latency above 2.5 s were significantly greater during alcohol sessions. **(G)** The percentage of latencies greater than 2.5 s were much higher during alcohol sessions. HP reward latencies over 2.5 s occurred significantly more during the second half of **(H)** alcohol, but not **(I)** SM sessions. LP reward latencies were similar between the first and second half during **(J)** alcohol and **(K)** SM sessions. The number of trials per session half is 14 for HP-Alc, 35 for HP-SM, 13 for LP-Alc, and 32 for LP-SM. *n* = 24 for panel **(A–D)**, *n* = 48 for panel **(E–G)**, *n* = 17–21/group for panel **(H–K)**. *p < 0.05, **p < 0.01 for group effect. All data are expressed as ± standard error mean. ^†^removed one outlier (over 200 s).

Reward latency, the time from giving a correct response to entering the reward tray, is a critical metric thought to identify motivation for the reward, with faster latency taken to indicate higher drive (Asinof and Paine, 2014). However, our initial analyses found that reward latency was not different between HP and LP mice during early-stage (Figure 3A, HPvLP: *F*_1,46_ = 0.9441, *p* = 0.3363) or late-stage (Figure 3B, HPvLP: *F*_1,46_ = 12.00, *p* = 0.2790) sessions for alcohol, or during strawberry milk sessions (Figure 3C, HPvLP: *F*_1,46_ = 0.0173, *p* = 0.8959). Furthermore, when averaging the reward latency of the final five ethanol sessions against the five SM sessions, reward latencies were significantly slower for alcohol compared with strawberry milk: HP mice had longer latencies for alcohol compared to HP responding for strawberry milk [HP-SM, Figure 3D, paired *t*-test, *t*(46) = 3.706, *p* = 0.0006]. Similarly, LP mice had longer latencies for alcohol compared to LP-SM [Figure 3D, paired *t*-test, *t*(45) = 3.719, *p* = 0.0006]. Also, HP and LP mice had similar reward latencies during SM testing [Figure 3D, student’s *t*-test, *t*(46) = 0.3285, *p* = 0.7440].

To better understand potential differences in reward latency, we examined the distribution of such latencies. Indeed, we found that alcohol reward latencies could be separated into several time intervals, those more similar to SM, and others that were much longer. Specifically, when comparing average latencies under 2.5 s, latency length was similar and quick when responding for alcohol or SM [Figure 3E, paired *t*-test, *t*(90) = 0.6917, *p* = 0.4909]. In contrast, the average length of reward latency above 2.5 s was significantly greater during alcohol testing [Figure 3F, paired *t*-test, *t*(90) = 2.666, *p* = 0.0091]. There were also significantly more longer-latency (> 2.5 s) responses during alcohol versus SM sessions [Figure 3G, paired *t*-test, *t*(94) = 3.794, *p* = 0.0003]. To better understand these longer latencies across a session, we identified whether they occurred in the first half or second half of a session (relative to number of completed trials). HP mice had significantly more longer latencies during the second half of the session for alcohol (Figure 3H, 1st-v-2nd-half: *F*_1,38_ = 6.318, *p* = 0.0163), while LP mice did not (Figure 3I, 1st-v-2nd-half: *F*_1,32_ = 0.1731, *p* = 0.6801). In addition, longer-latency responses for SM were fewer than for alcohol for HP and were equally distributed throughout the session in HP mice (Figure 3J, 1st-v-2nd-half: *F*_1,38_ = 1.332, *p* = 0.2556) and LP mice (Figure 3K, 1st-v-2nd-half: *F*_1,32_ = 0.243, *p* = 0.089). Cumulatively, these data suggest that HP mice exhibited a decrease in motivation for alcohol in the second half of the session, perhaps where mice getting more alcohol within the 5-CSRTT task participate less later in the session (addressed further in section “Responses for alcohol, not strawberry milk, in high-preference mice occur more within the first half of a session” and Supplementary discussion).

When reward latencies for alcohol were examined by consumption, HC and LC mice were not different during early-stage (Supplementary Figure 2A5, HCvLC: *F*_1,46_ = 0.0213, *p* = 0.8846), late-stage (Supplementary Figure 2B5, HCvLC: *F*_1,46_ = 0.1225, *p* = 0.7280; time: *F*_6_._30,289_._3_ = 2.159, *p* = 0.0441), or during strawberry milk sessions (Supplementary Figure 2C5, HCvLC: *F*_1,46_ = 0.1658, *p* = 0.6858). Since sorting the mice by preference proved to be more sensitive toward overall performance, consumption analysis was largely discontinued at this point.

### Responses for alcohol, not strawberry milk, in high-preference mice occur more within the first half of a session

As noted in section “Reward latency is increased during alcohol sessions, but not in strawberry milk, due to occasional longer reward latency trials,” changes in responding across a session may indicate altered drive for reward, e.g., where time-related shifts in reward latency in Figure 3 might relate to satiety, and such motivational changes across a session could be expressed in other measures such as less responding and/or less accurate responding. Representative trial-by-trial sessions visually describe clear differences in performance based on preference and reward (alcohol or SM, Supplementary Figure 7). Thus, we investigated differences in correct, incorrect, and omissions in the first versus second half of each session to confirm any behavioral shifts. Mice that averaged at least 10 correct responses across the last five alcohol late-stage training sessions were included in this analysis, in order to more clearly assess time-related changes in performance for alcohol or SM.

High-preference mice displayed significantly more correct responses in the first half versus second half of sessions (Supplementary Figure 6A1, 1st-v-2nd-half: *F*_1,38_ = 9.025, *p* = 0.0047) whereas LP mice had similar correct responses in both halves (Supplementary Figure 6A2, 1st-v-2nd-half: *F*_1,32_ = 0.6845, *p* = 0.4142). In contrast, the number of incorrect responses were similar between the first and second halves for both HP mice (Supplementary Figure 6C1, 1st-v-2nd-half: *F*_1,38_ = 0.0008, *p* = 0.9773) and LP mice (Supplementary Figure 6C2, 1st-v-2nd-half: *F*_1,32_ = 0.6345, *p* = 0.4316; time: *F*_3_._56,114_ = 4.254, *p* = 0.0043). In addition, while there were fewer overall omissions by later training, both HP (Supplementary Figure 6E1, 1st-v-2nd-half: *F*_1,38_ = 23.08, *p* < 0.0001) and LP (Supplementary Figure 6E2, 1st-v-2nd-half: *F*_1,32_ = 4.482, *p* = 0.0421) mice displayed higher omissions in the second half then the first half. In contrast to alcohol, during SM sessions there were no differences between first and second halves in correct responding in HP (Supplementary Figure 6B1, 1st-v-2nd-half: *F*_1,38_ = 1.232, *p* = 0.2739; time: *F*_3_._34,126_._9_ = 8.077, *p* < 0.0001) or LP mice (Supplementary Figure 6B2, 1st-v-2nd-half: *F*_1,32_ = 0.1309, *p* = 0.7199; time: *F*_2_._35,75_._24_ = 19.42, *p* < 0.0001), or in incorrect SM responses in HP (Supplementary Figure 6D1, 1st-v-2nd-half: *F*_1,38_ = 2.895, *p* = 0.0970; time: *F*_3_._38,128_._6_ = 6.412, *p* = 0.0002) or LP mice (Supplementary Figure 6D2, 1st-v-2nd-half: *F*_1,32_ = 2.195, *p* = 0.1483; time: *F*_2_._98,95_._44_ = 12.96, *p* < 0.0001). However, HP mice displayed more omissions in the second half (Supplementary Figure 6F1, 1st-v-2nd-half: *F*_1,38_ = 5.703, *p* = 0.0220; time: *F*_2_._833,107_._7_ = 4.249, *p* = 0.0081) and LP mice did not (Supplementary Figure 6F2, 1st-v-2nd-half: *F*_1,32_ = 2.195, *p* = 0.1483; time: *F*_2_._803,89_._71_ = 8.125, *p* = 0.0001; interaction: *F*_4,128_ = 2.507, *p* = 0.0453); however, the omissions difference in HP mice when responding for alcohol was *p* < 0.0001, while the comparable difference for SM was *p* = 0.0220. Together, these findings concur with reward latency results (Figure 3) that alcohol engagement in HP mice was greater during the first half of the session, which was overall not seen in LP mice or for SM responding in HP or LP, and we speculate that the second half decline in HP performance could reflect intoxicating effects of alcohol, satiety, or other factors (see Supplementary discussion).

### Intermittent alcohol exposure enhances behavioral engagement especially in previously low-engagement individuals

For Phase 2 of our studies, mice were allowed to drink alcohol under an Intermittent Access two-bottle choice (IA2BC) drinking paradigm, with 24-h access to 20% alcohol (versus water), three times a week, for 3 weeks. We were particularly interested in the possibility that IA2BC would not only enhance overall performance for alcohol, but specifically increase performance of LP mice. This could indicate that excessive consumption is particularly hazardous for individuals who innately have lower engagement with alcohol (while higher-engagement individuals already have greater risk for developing problem drinking).

Overall, HP and LP mice (defined by their alcohol behavior in initial DID sessions) had similar IA2BC consumption (Supplementary Figure 4O, HPvLP: *F*_1,46_ = 0.8358, *p* = 0.3654; time: *F*_3_._93,178_._3_ = 37.81, *p* < 0.0001) and preference (Supplementary Figure 4N, HPvLP: *F*_1,46_ = 3.373, *p* = 0.0728; time *F*_7_._81,355_._8_ = 11.25, *p* < 0.0001). However, HP did have greater preference during the first five sessions of IA2BC (Supplementary Figure 4N, HPvLP: *F*_1,46_ = 4.479, *p* = 0.0398; time *F*_3_._24,149_._1_ = 20.91, *p* < 0.0001), although consumption levels did not differ (Supplementary Figure 4O, HPvLP: *F*_1,46_ = 1.417, *p* = 0.2401; time *F*_1_._89,87_._01_ = 49.28, *p* < 0.0001).

**FIGURE 4:**
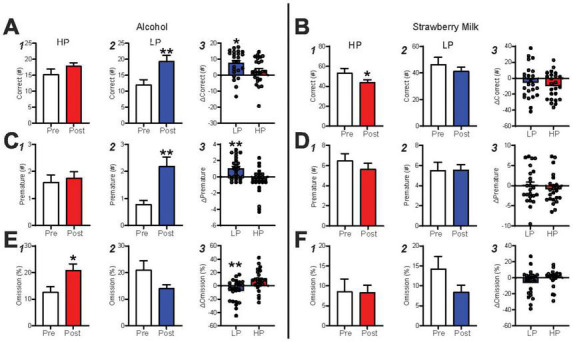
Intermittent alcohol exposure promotes responding in the 5-CSRTT. **(A)** LP mice gave more correct responses after IA2BC and had an overall greater change in correct than HP mice. **(B)** HP mice displayed a decrease in correct and had a similar change in performance as LP mice during SM sessions. **(C)** LP premature responding greatly increased post-IA2BC and had a greater performance change than HP mice, this was not seen during **(D)** SM sessions. **(E)** % Omissions were greater in HP post-IA2BC and LP mice had a greater performance change than HP mice. **(F)** No differences in % omission was found during SM sessions. *n* = 24/group, *p < 0.05, **p < 0.01 for effect of group. All data are expressed as ± standard error mean.

To better assess how IA2BC drinking might influence responding in HP and LP mice, we averaged measures in the last 3 late-stage alcohol sessions and compared them with the average of the last 3 post-IA2BC sessions. While pre-IA2BC versus post-IA2BC correct responses were not different for HP (Figure 4A1, *p* = 0.0995), IA2BC experience greatly increased correct responses in LP mice (Figure 4A2, *p* < 0.0001). In addition, the change in correct responses (pre versus post IA2BC) was significantly different between LP and HP mice (Figure 4A3, *U* = 176, *p* = 0.0202). In contrast, HP mice had lower correct responses during SM sessions (Figure 4B1, *p* = 0.0208), while LP mice did not (Figure 4B2, *p* = 0.2367), nor were there differences in relative change in correct (Figure 4B3, *p* = 0.4170). Thus, after IA2BC, LP showed significantly greater alcohol engagement, as indexed by number of correct responses, while HP mice showed less impact of IA2BC experience in this measure.

Similar to correct, premature responses for alcohol were unaffected by IA2BC in the HP (Figure 4C1, *p* = 0.6017) but were increased in LP (Figure 4C2, *p* = 0.0002) mice, with LP mice having significantly greater premature responding after IA2BC compared to HP (Figure 4C3, LP *U* = 141.5, *p* = 0.0019). SM premature were unaffected by IA2BC in HP (Figure 4D1, *p* = 0.2832) and LP (Figure 4D2, *p* = 0.9908), and with no differences in the change in performance after IA2BC between HP and LP (Figure 4D3, student’s *t*-test, t(46) = 0.7155, *p* = 0.4779). While premature responding can indicate increased impulsivity, we speculate that increased number of premature actions may occur in parallel with overall greater responding in the task, i.e., that premature responses may also reflect the level of behavioral engagement (and that increased premature in LP mice after IA2BC emphasizes their newfound readiness to respond for alcohol compared to pre-IA2BC, see section “Discussion”).

Finally, IA2BC did increase omissions for HP (Figure 4E1, *p* = 0.0126), with the opposite trend for LP (Figure 4E2, *p* = 0.1434), and LP had less loss of engagement for alcohol relative to HP [Figure 4E3, student’s *t*-test, *t*(46) = 3.24, *p* = 0.0022], indexed by these omissions. No changes were found in HP (Figure 4F1, *p* = 0.2076) or LP (Figure 4F2, *p* = 0.2182) during SM sessions, and relative performance changes were also similar between the groups (Figure 4F3, *U* = 208, *p* = 0.1015). Additional measures are detailed in Supplementary results and Supplementary Figure 8.

Together, these data strongly suggest that IA2BC overall increased engagement with alcohol responding significantly more in LP versus HP mice. Interestingly, in LP mice, IA2BC promoted responding even in mice who, pre-IA2BC, had barely responded (5 or fewer correct pre-IA2BC, increasing to ∼15 correct post-IA2BC). IA2BC also had limited impact on SM sessions, with only HP mice displaying decreased correct SM responses (although no decrease in alcohol responses). However, this SM decrease may also suggest decreased sucrose seeking behaviors after intermittent alcohol exposure as seen in previous studies (Starski et al., 2020). The effect of intermittent alcohol exposure on performance, regardless of preference, opens many avenues of investigation.

## Discussion

Excessive alcohol consumption is a widely prevalent activity that may promote the development of AUD, where alcohol intake becomes a necessity for the individual and becomes a considerable barrier to treatment. Increased motivation and abnormal attentiveness for alcohol are often signs of AUD, and individuals with higher trait impulsivity are also at greater risk for AUD (see section “Introduction”). Thus, it is critical, when seeking to develop novel treatments, to discover biological mechanisms that promote these forms of behavioral engagement for alcohol. In the current study, we used the 5-CSRTT, a behavioral paradigm that can measure a number of facets of behavioral engagement (e.g., attention, impulsivity, and motivation) in the same session. Importantly, we, for the first time, adopted the 5-CSRTT to train mice to respond for alcohol as reward. All previous studies examined how alcohol exposure alters 5-CSRTT responding for sugar, but we wanted the mice to associate the task with alcohol, rather than sugar, so that future implementation of more challenging forms of the task (e.g., to assess impulsivity under variable timing), will reflect their motivation and overall performance for alcohol. Interestingly, using this novel alcohol 5-CSRTT paradigm, we found that alcohol preference was a more sensitive indicator of performance for alcohol in the 5-CSRTT, rather than consumption. HP mice learned the task faster and had greater participation than LP mice. Further differences were found within sessions, where HP mice had more correct responses and faster latencies in the first half of the session, perhaps suggesting a form of “front-loading” behavior and/or satiety later in the session (see below). “Front-loading” is typically observed in alcohol drinking paradigms where the majority of intake occurs within a short period, usually 30 min, of initial alcohol access (Rhodes et al., 2007; Griffin et al., 2009; Barkley-Levenson and Crabbe, 2012; Maphis et al., 2022; Wilcox M. V. et al., 2014). In the current study, we contextualize front-loading as more correct responding within the first half of the session as the total session time is 30 min. Finally, we found that 3 weeks of alcohol drinking (IA2BC) greatly promoted responding for alcohol in LP mice, with lesser or no change in HP related to IA2BC. This suggests that IA2BC produced a greater increase in motivation for alcohol in mice that innately began with lower preference, while innately higher preference mice were already more alcohol-responsive. Importantly, IA2BC overall had little effect on SM sessions, further underscoring the specificity in IA2BC effects on increasing alcohol engagement in LP individuals.

Studies have used the 5-CSRTT to compare treatment effects on motivation, attention, and impulsivity for sugar (Torregrossa et al., 2012; Fletcher et al., 2013). Indeed, alcohol vapor, liquid diet, and gavage treatment have all been used to induce increase premature responding or attentional errors in the 5-CSRTT when responding for sugar (Semenova, 2012; Irimia et al., 2015; Broos et al., 2017; Starski et al., 2020). However, to date there are no studies we are aware of that exclusively use an intoxicant as reward for 5-CSRTT. One goal of this study was to effectively train mice in this complex task for an alcohol-only reward (10% alcohol), with no other additives (such as saccharin or even saccharin fade). This important advancement has allowed us to observe how motivated an animal was to wait during the intertrial interval, give a correct response, then retrieve the alcohol. Previous studies have trained animals to respond for alcohol in other behaviors such as differential reinforcement of low rates of responding (DRL) and progressive ratio tasks (Deehan et al., 2011; Somkuwar et al., 2018). While progressive ratio is valuable for measuring motivation, and DRL for impulsivity, the 5-CSRTT is designed to assess a broader range of measures in the same session. However, training animals in the 5-CSRTT with alcohol remains largely unexplored. We detail the nuances that come with having an intoxicant as a behavioral reward in this task in the Supplementary discussion, potential effects of increasing intoxication. Thus, we suggest that 5-CSRTT with alcohol as the reward can be robustly studied. This method of alcohol responding will likely be invaluable for future studies of how different interacting factors lead to different pathways to excessive drinking, including conditions with higher challenge (e.g., requiring greater attention or waiting) that some variants of 5-CSRTT testing can examine.

Both preference and consumption analyses revealed differences between their respective High/Low groups during early-stage sessions, however only preference analyses yielded consistent differences in number of trials and correct responses whereas all differences found during early-stage sessions disappeared during late-stage. Thus, we find that 5-CSRTT performance was significantly related to alcohol preference rather than consumption, including where HP mice demonstrated significantly more engagement with alcohol compared with LP mice. One speculation is that preference is related to an innate attention to and engagement with some condition. In this model, HP mice may reflect an individual who repeatedly orders a drink when there is an inherent waiting period (bartender order to delivery of alcohol), payment (correct response), and, finally, reward retrieval (consumption of drink). LP mice, however, may be compared with a more social drinker, where they drink when alcohol is easily available (two-bottle choice), but are more likely to be dissuaded if it requires actively work for it (purchasing, waiting, or traveling for alcohol). To further speculate, it is interesting that, in addition to high trait impulsivity, people at risk for binging have higher alcohol preference (Poulos et al., 1995; Crews and Boettiger, 2009; Schumann et al., 2011). However, determining clear relations in human studies remains challenging, and the ability to control factors in rodent studies has given insights into the alcohol-impulsivity relationship. For example, mice genetically selected for high alcohol preference display higher impulsivity in a delay discounting task compared with mice selected for low preference, although some aspects of impulsivity (amphetamine and lithium reduction of impulsivity) are not related to preference (Oberlin et al., 2010; Halcomb et al., 2013). In addition, mice genetically selected for high alcohol consumption displayed impaired response inhibition in a Go/No-go task but were not different from low consumption in delay discounting (Wilhelm et al., 2007). However, we should note that consumption level is still a very important factor, and several studies have assessed differential neural mechanisms that underlie higher versus lower intake level (Juarez et al., 2019; Lei et al., 2019). We recently performed BECs in C57BL/6 mice during limited access alcohol paradigms, and they reach binge level on average of 1.6 g/kg for a 30 min session for 20% alcohol (Lei et al., 2016; Wegner et al., 2017; Kwok et al., 2021), this amount approximates 85–100 mg% BEC. Mice in this study had access to roughly 1.3–1.5 g/kg of 10% alcohol which the mice may reach approximately 70–80 mg% BEC. This BEC level is near the 100 mg% that suggests behavioral changes from intoxication (Crabbe, 2012). It is necessary in future studies to strategically collect BEC levels in mice shortly after a behavioral session within our paradigm. Thus, we emphasize that HP and LP had similar drinking levels that, based on blood alcohol assessments in our previous mouse studies (Lei et al., 2016; Kwok et al., 2021), both HP and LP drank sufficient alcohol to on average reach binge level. In addition, premature responding in the 5-CSRTT is an indicator of impulsivity, and we found that HP mice had higher premature responses during early-stage training. This may suggest that their propensity for alcohol promotes error in the ability to wait (for the stimulus), although we speculate that greater premature responses along with greater overall responding could in some cases reflect greater engagement rather than impulsivity *per se*. Future studies will be needed to further dissect these and other aspects of behavior that promote excessive drinking. Cumulatively, the field continues to make substantial strides toward understanding the relationship between impulsivity, alcohol preference and intake, but the interconnection of these remains ambiguous.

Impulsivity is a complex construct that has come under scrutiny due to the wide breadth of behaviors it encompasses (Meda et al., 2009; MacKillop et al., 2016; Strickland and Johnson, 2021). Risk-taking tasks, delay-discounting, DRL, reversal learning, Go/No-Go, and, here, the 5-CSRTT are examples of impulsivity-related tasks, however they all measure impulsivity in different ways. Impulsive behaviors have traditionally been separated into two domains, impulsive action and Impulsive choice (Winstanley et al., 2006; Perry and Carroll, 2008; Winstanley, 2011; Jentsch et al., 2014). Under impulsive action, Go/No-Go and reversal learning measure action inhibition while the 5-CSRTT measures the ability to wait. In contrast, impulsive choice tasks measure sensitivity to delayed (e.g., delay-discounting) and risky choices (e.g., Balloon analogue risk task). We have chosen the 5-CSRTT since the task allows assessment of premature responses (related to impulsivity) as well as omissions and accuracy (more clear indicators of engagement). However, premature responses during standard training sessions may describe participation opposed to impulsivity. Future studies will utilize randomized waiting periods to truly test impulsive responding. Thus, with 5-CSRTT we have the ability to measure impulsivity (among other measures) related to voluntary alcohol acquisition, especially in future (and ongoing) studies using 5-CSRTT variants that more clearly assess (e.g., variable timing of reward presentation). Importantly, even with the broad nature of impulsivity, clinical studies using impulsivity tasks often shown its relationship to alcohol use (Dick et al., 2010; Lejuez et al., 2010; Adams et al., 2013; Elder et al., 2019; Herman and Duka, 2019) and the 5-CSRTT has been used clinically to predict higher alcohol intake in highly impulsive individuals (Sanchez-Roige et al., 2014a). Here, we attempted to bridge a much-needed gap in rodent alcohol-impulsivity studies so that future work can focus on impulsive action for voluntary alcohol consumption, and related behavioral indicators of excessive intake, to decipher potential patterns and biomarkers that mirror clinical findings.

Here we focus on mice that have reached adulthood and continued to observe their progression toward behavioral engagement for alcohol. In humans, the average age of first drink is just over 17 (Caetano et al., 2014), which is approximately equivalent to the age of the mice at inception of the experiment (8 weeks) (Dutta and Sengupta, 2016; Wang et al., 2020), thus we are able to observe the motivation of a translational timepoint in which individuals begin to drink. In addition, while we did not explicitly examine age, other important and interesting studies have shown that intermittent alcohol exposure starting during earlier adolescence can have stronger changes in affect- and motivation-related behavior than alcohol-exposed adults (Van Skike et al., 2015; Kim et al., 2019; Nentwig et al., 2019; Healey et al., 2022). Here, using the intermittent access paradigm, we described a stark change in lower preference, less alcohol-engaged mice, where intermittent alcohol was related to a behavior shift toward higher alcohol responding and more impulsivity. Thus, these mice might model a lower trait risk in humans, which nonetheless can be shifted to more maladaptive responding with sufficient alcohol drinking. It is also important to note that, during adolescence, animal models involving repeated alcohol exposure (alcohol vapor) find increased impulsivity and decreased attention within the 5-CSRTT, in addition to other cognitive behavioral problems (Coleman et al., 2011, 2014; Semenova, 2012; Seemiller and Gould, 2020). Thus, it remains to be determined whether the same aspect of adolescence is a factor in own studies.

A limitation of this study is the inclusion of only male mice since female mice may present differences in alcohol engagement within the 5-CSRTT. Recently, the number of women with problem alcohol drinking has increased and these problems can be more severe (Brady and Randall, 1999; Erol and Karpyak, 2015; White et al., 2015; Becker and Koob, 2016; Grant et al., 2017; Carvalho et al., 2019). E.g., women can exhibit greater deficits in inhibitory control as a result from heavy drinking, and comorbidities, such as stress, promote drinking behavior more than in men (Peltier et al., 2019). Further, women with a family history of AUD exhibit higher error in go/no-go tasks (Saunders et al., 2008; Nederkoorn et al., 2009; Weafer et al., 2015). Sex differences in delay discounting tasks have conflicting reports (Beck and Triplett, 2009; Weafer and de Wit, 2014), however it was observed that women discounted alcohol more compared with monetary rewards than males (Yankelevitz et al., 2012). In rodent studies, sex differences have been found in the modulation of noradrenaline on attention and impulse control where a noradrenaline reuptake inhibitor was more effective in reducing impulsivity in male rats than female (Mei et al., 2021). Alcohol drinking and impulsive action behavior was found to be strongly associated only in male rats (Hammerslag et al., 2019). Additionally, transgenic BDNF changes induce female-only changes in impulsivity in 5-CSRTT and alcohol self-administration (Hogan et al., 2021). Thus, potential sex differences in mechanisms that underlie impulsivity, behavioral engagement for alcohol, as well as related divergences in underlying cortical circuitry and motivation drives (Barker and Taylor, 2019; Flores-Bonilla and Richardson, 2020), make it critical to use both sexes in future studies.

Several studies have shown how disruptions in various cortical areas can alter behavioral engagement detected through the 5-CSRTT (Chudasama et al., 2003; Pisansky et al., 2019; Starski et al., 2019). The anterior cingulate cortex (ACC), a region involved in higher-level cognitive function, modulates accuracy and omissions (Chudasama et al., 2003; Norman et al., 2021) and is required for top-down action control within the 5-CSRTT (White et al., 2018). Further, errors in decision-making after chronic alcohol use have been linked to ACC (Mashhoon et al., 2014; Smith et al., 2017; Zakiniaeiz et al., 2017). Additionally, the anterior insula (aINS) has been implicated in impulsive behaviors (Kayser et al., 2012; Pattij et al., 2014; Centanni et al., 2021), including where premature responding in the 5-CSRTT correlates with aINS thinning in rat (Belin-Rauscent et al., 2016). The aINS has also been heavily associated with attention (Rolls, 2016; Haaranen et al., 2020a,b; Happer et al., 2021), as a critical part of the salience network, and been implicated in problem alcohol drinking (Menon and Uddin, 2010; Cardenas et al., 2011; Seif et al., 2013; Grodin et al., 2018; Campbell et al., 2019). Further, motivational aspects of alcohol have been strongly linked with particular brain circuits including nucleus accumbens dopamine and glutamate signaling (Brodie et al., 1990; Gonzales and Weiss, 1998; Spiga et al., 2014; Sutera et al., 2016; Brancato et al., 2017, 2021) and future work is required to examine the brain mechanisms underlying the responding for alcohol we examine here. Though there are many regions associated with behavioral engagement, the ACC and aINS may be future targets of investigation linking intermittent alcohol intake and changes in engagement for alcohol.

Currently, investigators may be hesitant to replace the typical appetitive reward with an intoxicant. However, in the present study, we observed the satisfactory performance of mice while training in the 5-CSRTT for an alcohol reward. This paradigm displays the relationship between motivation for freely accessible alcohol (DID, two-bottle choice) and for when mice must perform an attention-based task (5-CSRTT) for alcohol. Interestingly, HP mice were more likely to perform the task than their LP counterparts, while protracted alcohol consumption increased engagement with alcohol in LP mice significantly more than HP, while SM responding was higher than for alcohol and overall not different in HP versus LP or before and after IA2BC. In addition, differences in performance found within HP sessions emphasize the importance of doing a trial-by-trial analysis to maximize the efficacy and interpretability of the data. Though we do not have a non-IA2BC control, the differences between HP and LP mice after IA2BC found within this study detail the risk intermittent alcohol intake has on problem drinking even in those that did not prefer alcohol. Thus, our behaviorally focused series of experiments set a foundation to answer important neurological hypotheses that will be featured in future studies. Cumulatively, our findings offer a novel insight into preference-related motivation for and engagement with alcohol, that can be used to identify behavioral patterns and brain mechanisms among the different factors (including attention, reward motivation, impulsivity, and perseveration) that could come together in different ways to promote excessive alcohol drinking and its substantial harms.

## Data availability statement

The raw data supporting the conclusions of this article will be made available upon request to the authors, without undue reservation.

## Ethics statement

The animal study was reviewed and approved by the Indiana University Institutional Animal Care and Use Committee.

## Author contributions

PS, DM, and HM designed the behavioral schedule and collected the data. PS and DM analyzed the data. PS and FH wrote the manuscript. All authors provided critical review of the content and approved of the final version for publication.

## References

[B1] Adams Z. W. Milich R. Lynam D. R. Charnigo R. J. (2013). Interactive effects of drinking history and impulsivity on college drinking. *Addict. Behav.* 38 2860–2867. 10.1016/j.addbeh.2013.08.009 24018231 PMC4075284

[B2] Asinof S. K. Paine T. A. (2014). The 5-choice serial reaction time task: A task of attention and impulse control for rodents. *J. Vis. Exp.* 90:e51574. 10.3791/51574 25146934 PMC6592595

[B3] Bari A. Dalley J. W. Robbins T. W. (2008). The application of the 5-choice serial reaction time task for the assessment of visual attentional processes and impulse control in rats. *Nat. Protoc.* 3 759–767. 10.1038/nprot.2008.41 18451784

[B4] Barker J. M. Taylor J. R. (2019). Sex differences in incentive motivation and the relationship to the development and maintenance of alcohol use disorders. *Physiol. Behav.* 203 91–99. 10.1016/j.physbeh.2017.09.027 28974459 PMC5878117

[B5] Barkley-Levenson A. M. Crabbe J. C. (2012). Ethanol drinking microstructure of a high drinking in the dark selected mouse line. *Alcohol. Clin. Exp. Res.* 36 1330–1339. 10.1111/j.1530-0277.2012.01749.x 22524154 PMC3407303

[B6] Beck R. C. Triplett M. F. (2009). Test-retest reliability of a group-administered paper-pencil measure of delay discounting. *Exp. Clin. Psychopharmacol.* 17 345–355. 10.1037/a0017078 19803634

[B7] Becker J. B. Koob G. F. (2016). Sex differences in animal models: Focus on addiction. *Pharmacol. Rev.* 68 242–263. 10.1124/pr.115.011163 26772794 PMC4813426

[B8] Belin-Rauscent A. Daniel M. L. Puaud M. Jupp B. Sawiak S. Howett D. (2016). From impulses to maladaptive actions: The insula is a neurobiological gate for the development of compulsive behavior. *Mol. Psychiatry* 21 491–499. 10.1038/mp.2015.140 26370145

[B9] Brady K. T. Randall C. L. (1999). Gender differences in substance use disorders. *Psychiatr. Clin. North Am.* 22 241–252. 10.1016/S0193-953X(05)70074-5 10385931

[B10] Brancato A. Castelli V. Lavanco G. Tringali G. Micale V. Kuchar M. (2021). Binge-like alcohol exposure in adolescence: Behavioural, Neuroendocrine and molecular evidence of abnormal neuroplasticity. and return. *Biomedicines* 9:1161. 10.3390/biomedicines9091161 34572345 PMC8470908

[B11] Brancato A. Lavanco G. Cavallaro A. Plescia F. Cannizzaro C. (2017). ’Acetaldehyde, motivation and stress: Behavioral evidence of an addictive menage a trois. *Front. Behav. Neurosci.* 11:23. 10.3389/fnbeh.2017.00023 28232795 PMC5299001

[B12] Brodie M. S. Shefner S. A. Dunwiddie T. V. (1990). Ethanol increases the firing rate of dopamine neurons of the rat ventral tegmental area in vitro. *Brain Res.* 508 65–69. 10.1016/0006-8993(90)91118-Z 2337793

[B13] Broos N. van Mourik Y. Schetters D. De Vries T. J. Pattij T. (2017). Dissociable effects of cocaine and yohimbine on impulsive action and relapse to cocaine seeking. *Psychopharmacology (Berl)* 234 3343–3351. 10.1007/s00213-017-4711-9 28856391 PMC5660838

[B14] Caetano R. Mills B. A. Vaeth P. A. Reingle J. (2014). Age at first drink, drinking, binge drinking, and DSM-5 alcohol use disorder among Hispanic national groups in the United States. *Alcohol. Clin. Exp. Res.* 38 1381–1389. 10.1111/acer.12354 24689445 PMC4063311

[B15] Campbell E. J. Flanagan J. P. M. Walker L. C. Hill M. K. Marchant N. J. Lawrence A. J. (2019). Anterior insular cortex is critical for the propensity to relapse following punishment-imposed abstinence of alcohol seeking. *J. Neurosci.* 39 1077–1087. 10.1523/JNEUROSCI.1596-18.2018 30509960 PMC6363928

[B16] Cardenas V. A. Durazzo T. C. Gazdzinski S. Mon A. Studholme C. Meyerhoff D. J. (2011). Brain morphology at entry into treatment for alcohol dependence is related to relapse propensity. *Biol. Psychiatry* 70 561–567. 10.1016/j.biopsych.2011.04.003 21601177 PMC3162109

[B17] Carvalho A. F. Heilig M. Perez A. Probst C. Rehm J. (2019). Alcohol use disorders. *Lancet* 394 781–792. 10.1016/S0140-6736(19)31775-1 31478502

[B18] CDC (2014). *Excessive drinking costs U.S. $223.5 Billion.* Atlanta, GA: Center for Disease Control.

[B19] Centanni S. W. Janes A. C. Haggerty D. L. Atwood B. Hopf F. W. (2021). Better living through understanding the insula: Why subregions can make all the difference. *Neuropharmacology* 198:108765. 10.1016/j.neuropharm.2021.108765 34461066 PMC13284909

[B20] Chudasama Y. Passetti F. Rhodes S. E. Lopian D. Desai A. Robbins T. W. (2003). Dissociable aspects of performance on the 5-choice serial reaction time task following lesions of the dorsal anterior cingulate, infralimbic and orbitofrontal cortex in the rat: Differential effects on selectivity, impulsivity and compulsivity. *Behav. Brain Res.* 146 105–119. 10.1016/j.bbr.2003.09.020 14643464

[B21] Coleman L. G. Jr. He J. Lee J. Styner M. Crews F. T. (2011). Adolescent binge drinking alters adult brain neurotransmitter gene expression, behavior, brain regional volumes, and neurochemistry in mice. *Alcohol. Clin. Exp. Res.* 35 671–688. 10.1111/j.1530-0277.2010.01385.x 21223304 PMC3544413

[B22] Coleman L. G. Jr. Liu W. Oguz I. Styner M. Crews F. T. (2014). Adolescent binge ethanol treatment alters adult brain regional volumes, cortical extracellular matrix protein and behavioral flexibility. *Pharmacol. Biochem. Behav.* 116 142–151. 10.1016/j.pbb.2013.11.021 24275185 PMC3913047

[B23] Crabbe J. C. (2012). Translational behaviour-genetic studies of alcohol: Are we there yet? *Genes. Brain. Behav.* 11, 375–386. 10.1111/j.1601-183X.2012.00798.x 22510368 PMC3370065

[B24] Crews F. T. Boettiger C. A. (2009). Impulsivity, frontal lobes and risk for addiction. *Pharmacol. Biochem. Behav.* 93 237–247. 10.1016/j.pbb.2009.04.018 19410598 PMC2730661

[B25] Dawson D. A. Grant B. F. Li T. K. (2005). Quantifying the risks associated with exceeding recommended drinking limits. *Alcohol. Clin. Exp. Res.* 29 902–908. 10.1097/01.ALC.0000164544.45746.A7 15897737

[B26] Deehan G. A. Jr. Palmatier M. I. Cain M. E. Kiefer S. W. (2011). Differential rearing conditions and alcohol-preferring rats: Consumption of and operant responding for ethanol. *Behav. Neurosci.* 125 184–193. 10.1037/a0022627 21280936

[B27] Dick D. M. Smith G. Olausson P. Mitchell S. H. Leeman R. F. O’Malley S. S. (2010). Understanding the construct of impulsivity and its relationship to alcohol use disorders. *Addict. Biol.* 15 217–226. 10.1111/j.1369-1600.2009.00190.x 20148781 PMC2895996

[B28] Dutta S. Sengupta P. (2016). Men and mice: Relating their ages. *Life Sci.* 152 244–248. 10.1016/j.lfs.2015.10.025 26596563

[B29] Elder J. Brieant A. Lauharatanahirun N. King-Casas B. Kim-Spoon J. (2019). Insular risk processing predicts alcohol use via externalizing pathway in male adolescents. *J. Stud. Alcohol. Drugs* 80 602–613. 10.15288/jsad.2019.80.602 31790350 PMC6900996

[B30] Erol A. Karpyak V. M. (2015). Sex and gender-related differences in alcohol use and its consequences: Contemporary knowledge and future research considerations. *Drug Alcohol. Depend.* 156 1–13. 10.1016/j.drugalcdep.2015.08.023 26371405

[B31] Esser M. B. Hedden S. L. Kanny D. Brewer R. D. Gfroerer J. C. Naimi T. S. (2014). Prevalence of alcohol dependence among US adult drinkers, 2009-2011. *Prev. Chronic Dis.* 11:E206. 10.5888/pcd11.140329 25412029 PMC4241371

[B32] Fadardi J. S. Cox W. M. (2006). Alcohol attentional bias: Drinking salience or cognitive impairment? *Psychopharmacology (Berl)* 185 169–178. 10.1007/s00213-005-0268-0 16491429

[B33] Fletcher P. J. Soko A. D. Higgins G. A. (2013). Impulsive action in the 5-choice serial reaction time test in 5-HT(2)c receptor null mutant mice. *Psychopharmacology (Berl)* 226 561–570. 10.1007/s00213-012-2929-0 23192316

[B34] Flores-Bonilla A. Richardson H. N. (2020). Sex Differences in the neurobiology of alcohol use disorder. *Alcohol Res.* 40:04. 10.35946/arcr.v40.2.04 33042719 PMC7532022

[B35] Gonzales R. A. Weiss F. (1998). Suppression of ethanol-reinforced behavior by naltrexone is associated with attenuation of the ethanol-induced increase in dialysate dopamine levels in the nucleus accumbens. *J. Neurosci.* 18 10663–10671. 10.1523/JNEUROSCI.18-24-10663.1998 9852601 PMC6793337

[B36] Gowin J. L. Sloan M. E. Stangl B. L. Vatsalya V. Ramchandani V. A. (2017). Vulnerability for alcohol use disorder and rate of alcohol consumption. *Am. J. Psychiatry* 174 1094–1101. 10.1176/appi.ajp.2017.16101180 28774194 PMC5667663

[B37] Grant B. F. Chou S. P. Saha T. D. Pickering R. P. Kerridge B. T. Ruan W. J. (2017). Prevalence of 12-month alcohol use, high-risk drinking, and DSM-IV alcohol use disorder in the United States, 2001-2002 to 2012-2013: Results from the national epidemiologic survey on alcohol and related conditions. *JAMA Psychiatry* 74 911–923. 10.1001/jamapsychiatry.2017.2161 28793133 PMC5710229

[B38] Grant B. F. Goldstein R. B. Saha T. D. Chou S. P. Jung J. Zhang H. (2015). Epidemiology of DSM-5 alcohol use disorder: Results from the national epidemiologic survey on alcohol and related conditions III. *JAMA Psychiatry* 72 757–766. 10.1001/jamapsychiatry.2015.0584 26039070 PMC5240584

[B39] Griffin W. C. III Lopez M. F. Becker H. C. (2009). Intensity and duration of chronic ethanol exposure is critical for subsequent escalation of voluntary ethanol drinking in mice. *Alcohol. Clin. Exp. Res.* 33 1893–1900. 10.1111/j.1530-0277.2009.01027.x 19673744 PMC2995298

[B40] Grodin E. N. Sussman L. Sundby K. Brennan G. M. Diazgranados N. Heilig M. (2018). Neural correlates of compulsive alcohol seeking in heavy drinkers. *Biol. Psychiatry Cogn. Neurosci. Neuroimaging* 3 1022–1031. 10.1016/j.bpsc.2018.06.009 30143454

[B41] Haaranen M. Schafer A. Jarvi V. Hyytia P. (2020a). Chemogenetic Stimulation and silencing of the insula, amygdala, nucleus accumbens, and their connections differentially modulate alcohol drinking in rats. *Front. Behav. Neurosci.* 14:580849. 10.3389/fnbeh.2020.580849 33328918 PMC7671963

[B42] Haaranen M. Scuppa G. Tambalo S. Jarvi V. Bertozzi S. M. Armirotti A. (2020b). Anterior insula stimulation suppresses appetitive behavior while inducing forebrain activation in alcohol-preferring rats. *Transl. Psychiatry* 10:150. 10.1038/s41398-020-0833-7 32424183 PMC7235223

[B43] Halcomb M. E. Gould T. D. Grahame N. J. (2013). Lithium, but not valproate, reduces impulsive choice in the delay-discounting task in mice. *Neuropsychopharmacology* 38 1937–1944. 10.1038/npp.2013.89 23584261 PMC3746699

[B44] Hammerslag L. R. Belagodu A. P. Aladesuyi Arogundade O. A. Karountzos A. G. Guo Q. Galvez R. (2019). Adolescent impulsivity as a sex-dependent and subtype-dependent predictor of impulsivity, alcohol drinking and dopamine D2 receptor expression in adult rats. *Addict. Biol.* 24 193–205. 10.1111/adb.12586 29210144 PMC5988863

[B45] Happer J. P. Wagner L. C. Beaton L. E. Rosen B. Q. Marinkovic K. (2021). The “when” and “where” of the interplay between attentional capture and response inhibition during a Go/NoGo variant. *Neuroimage* 231:117837. 10.1016/j.neuroimage.2021.117837 33577939 PMC8154719

[B46] Healey K. L. Kibble S. A. Bell A. Kramer G. Maldonado-Devincci A. Swartzwelder H. S. (2022). Sex differences in the effects of adolescent intermittent ethanol exposure on exploratory and anxiety-like behavior in adult rats. *Alcohol* 98 43–50. 10.1016/j.alcohol.2021.11.002 34808302 PMC8714675

[B47] Heitmann J. Jonker N. C. Ostafin B. D. de Jong P. J. (2020). Attentional bias for alcohol cues in visual search-Increased engagement, difficulty to disengage or both? *PLoS One* 15:e0228272. 10.1371/journal.pone.0228272 31986192 PMC6984682

[B48] Herman A. M. Duka T. (2019). Facets of impulsivity and alcohol use: What role do emotions play? *Neurosci. Biobehav. Rev.* 106 202–216. 10.1016/j.neubiorev.2018.08.011 30343823

[B49] Hogan N. L. Jaehne E. J. Bak S. Djouma E. van den Buuse M. (2021). Brain-derived neurotrophic factor Val66Met induces female-specific changes in impulsive behaviour and alcohol self-administration in mice. *Behav. Brain Res.* 401:113090. 10.1016/j.bbr.2020.113090 33358916

[B50] Hwa L. S. Chu A. Levinson S. A. Kayyali T. M. DeBold J. F. Miczek K. A. (2011). Persistent escalation of alcohol drinking in C57BL/6J mice with intermittent access to 20% ethanol. *Alcohol. Clin. Exp. Res.* 35 1938–1947. 10.1111/j.1530-0277.2011.01545.x 21631540 PMC3166538

[B51] Irimia C. Wiskerke J. Natividad L. A. Polis I. Y. de Vries T. J. Pattij T. (2015). Increased impulsivity in rats as a result of repeated cycles of alcohol intoxication and abstinence. *Addict. Biol.* 20 263–274. 10.1111/adb.12119 24341858 PMC4061283

[B52] Jentsch J. D. Ashenhurst J. R. Cervantes M. C. Groman S. M. James A. S. Pennington Z. T. (2014). Dissecting impulsivity and its relationships to drug addictions. *Ann. N. Y. Acad. Sci.* 1327 1–26. 10.1111/nyas.12388 24654857 PMC4360991

[B53] Juarez B. Liu Y. Zhang L. Han M. H. (2019). Optogenetic investigation of neural mechanisms for alcohol-use disorder. *Alcohol* 74 29–38. 10.1016/j.alcohol.2018.05.005 30621856 PMC7581464

[B54] Kayser A. S. Allen D. C. Navarro-Cebrian A. Mitchell J. M. Fields H. L. (2012). Dopamine, corticostriatal connectivity, and intertemporal choice. *J. Neurosci.* 32 9402–9409. 10.1523/JNEUROSCI.1180-12.2012 22764248 PMC6622221

[B55] Kim E. U. Varlinskaya E. I. Dannenhoffer C. A. Spear L. P. (2019). Adolescent intermittent ethanol exposure: Effects on pubertal development, novelty seeking, and social interaction in adulthood. *Alcohol* 75 19–29. 10.1016/j.alcohol.2018.05.002 30326391

[B56] Kwok C. Lei K. Pedrozo V. Anderson L. Ghotra S. Walsh M. (2021). Differential importance of nucleus accumbens Ox1Rs and AMPARs for female and male mouse binge alcohol drinking. *Sci. Rep.* 11:231. 10.1038/s41598-020-79935-2 33420199 PMC7794293

[B57] Larimer M. E. Palmer R. S. Marlatt G. A. (1999). ’Relapse prevention. An overview of Marlatt’s cognitive-behavioral model. *Alcohol. Res. Health* 23 151–160. 10890810 PMC6760427

[B58] Lau-Barraco C. Linden-Carmichael A. N. Hequembourg A. Pribesh S. (2017). Motivations and consequences of alcohol use among heavy drinking nonstudent emerging adults. *J. Adolesc. Res.* 32 667–695. 10.1177/0743558416630812 29151670 PMC5684885

[B59] Lei K. Kwok C. Darevsky D. Wegner S. A. Yu J. Nakayama L. (2019). Nucleus accumbens shell orexin-1 receptors are critical mediators of binge intake in excessive-drinking individuals. *Front. Neurosci.* 13:88. 10.3389/fnins.2019.00088 30814925 PMC6381036

[B60] Lei K. Wegner S. A. Yu J. H. Hopf F. W. (2016). Orexin-1 receptor blockade suppresses compulsive-like alcohol drinking in mice. *Neuropharmacology* 110 431–437. 10.1016/j.neuropharm.2016.08.008 27523303 PMC5065938

[B61] Lejuez C. W. Magidson J. F. Mitchell S. H. Sinha R. Stevens M. C. de Wit H. (2010). Behavioral and biological indicators of impulsivity in the development of alcohol use, problems, and disorders. *Alcohol. Clin. Exp. Res.* 34 1334–1345. 10.1111/j.1530-0277.2010.01217.x 20491733 PMC3182265

[B62] MacKillop J. Weafer J. C Gray J. Oshri A. Palmer A. de Wit H. (2016). The latent structure of impulsivity: Impulsive choice, impulsive action, and impulsive personality traits. *Psychopharmacology (Berl)* 233 3361–3370. 10.1007/s00213-016-4372-0 27449350 PMC5204128

[B63] Maphis N. M. Huffman R. T. Linsenbardt D. N. (2022). The development, but not expression, of alcohol front-loading in C57BL/6J mice maintained on LabDiet 5001 is abolished by maintenance on Teklad 2920x rodent diet. *Alcohol. Clin. Exp. Res.* 46 1321–1330. 10.1111/acer.14876 35633038 PMC9357207

[B64] Mashhoon Y. Czerkawski C. Crowley D. J. Cohen-Gilbert J. E. Sneider J. T. Silveri M. M. (2014). ’Binge alcohol consumption in emerging adults: Anterior cingulate cortical “thinness” is associated with alcohol use patterns. *Alcohol. Clin. Exp. Res.* 38 1955–1964. 10.1111/acer.12475 24961871 PMC4107054

[B65] Meda S. A. Stevens M. C. Potenza M. N. Pittman B. Gueorguieva R. Andrews M. M. (2009). Investigating the behavioral and self-report constructs of impulsivity domains using principal component analysis. *Behav. Pharmacol.* 20 390–399. 10.1097/FBP.0b013e32833113a3 19724194 PMC3268653

[B66] Mei X. Wang L. Yang B. Li X. (2021). Sex differences in noradrenergic modulation of attention and impulsivity in rats. *Psychopharmacology (Berl)* 238 2167–2177. 10.1007/s00213-021-05841-8 33834255

[B67] Menon V. Uddin L. Q. (2010). Saliency, switching, attention and control: A network model of insula function. *Brain Struct. Funct.* 214 655–667. 10.1007/s00429-010-0262-0 20512370 PMC2899886

[B68] Metten P. Schlumbohm J. P. Huang L. C. Greenberg G. D. Hack W. R. Spence S. E. (2018). An alcohol withdrawal test battery measuring multiple behavioral symptoms in mice. *Alcohol* 68 19–35. 10.1016/j.alcohol.2017.08.014 29427828 PMC5839916

[B69] Monem R. Fillmore M. T. (2019). Alcohol administration reduces attentional bias to alcohol-related but not food-related cues: Evidence for a satiety hypothesis. *Psychol. Addict. Behav.* 33 677–684. 10.1037/adb0000522 31599605 PMC6888930

[B70] Moos R. H. Moos B. S. (2006). Rates and predictors of relapse after natural and treated remission from alcohol use disorders. *Addiction* 101 212–222. 10.1111/j.1360-0443.2006.01310.x 16445550 PMC1976118

[B71] Nederkoorn C. Baltus M. Guerrieri R. Wiers R. W. (2009). Heavy drinking is associated with deficient response inhibition in women but not in men. *Pharmacol. Biochem. Behav.* 93 331–336. 10.1016/j.pbb.2009.04.015 19409923

[B72] Nentwig T. B. Starr E. M. Chandler L. J. Glover E. J. (2019). Absence of compulsive drinking phenotype in adult male rats exposed to ethanol in a binge-like pattern during adolescence. *Alcohol* 79 93–103. 10.1016/j.alcohol.2019.01.006 30664983 PMC6639162

[B73] Norman K. J. Bateh J. Maccario P. Cho C. Caro K. Nishioka T. (2021). Frontal-sensory cortical projections become dispensable for attentional performance upon a reduction of task demand in mice. *Front. Neurosci.* 15:775256. 10.3389/fnins.2021.775256 35087372 PMC8787360

[B74] Oberlin B. G. Grahame N. J. (2009). High-alcohol preferring mice are more impulsive than low-alcohol preferring mice as measured in the delay discounting task. *Alcohol. Clin. Exp. Res.* 33 1294–1303. 10.1111/j.1530-0277.2009.00955.x 19389183 PMC2872785

[B75] Oberlin B. G. Bristow R. E. Heighton M. E. Grahame N. J. (2010). Pharmacologic dissociation between impulsivity and alcohol drinking in high alcohol preferring mice. *Alcohol. Clin. Exp. Res.* 34 1363–1375. 10.1111/j.1530-0277.2010.01220.x 20491739 PMC3967789

[B76] Pattij T. Schetters D. Schoffelmeer A. N. (2014). Dopaminergic modulation of impulsive decision making in the rat insular cortex. *Behav. Brain Res.* 270 118–124. 10.1016/j.bbr.2014.05.010 24837747

[B77] Peltier M. R. Verplaetse T. L. Mineur Y. S. Petrakis I. L. Cosgrove K. P. Picciotto M. R. (2019). Sex differences in stress-related alcohol use. *Neurobiol. Stress* 10:100149. 10.1016/j.ynstr.2019.100149 30949562 PMC6430711

[B78] Pena-Oliver Y. Giuliano C. Economidou D. Goodlett C. R. Robbins T. W. Dalley J. W. (2015). Alcohol-preferring rats show goal oriented behaviour to food incentives but are neither sign-trackers nor impulsive. *PLoS One* 10:e0131016. 10.1371/journal.pone.0131016 26098361 PMC4476783

[B79] Perry J. L. Carroll M. E. (2008). The role of impulsive behavior in drug abuse. *Psychopharmacology (Berl)* 200 1–26. 10.1007/s00213-008-1173-0 18600315

[B80] Pisansky M. T. Lefevre E. M. Retzlaff C. L. Trieu B. H. Leipold D. W. Rothwell P. E. (2019). Nucleus accumbens fast-spiking interneurons constrain impulsive action. *Biol. Psychiatry* 86 836–847. 10.1016/j.biopsych.2019.07.002 31471038 PMC6823148

[B81] Poulos C. X. Le A. D. Parker J. L. (1995). Impulsivity predicts individual susceptibility to high levels of alcohol self-administration. *Behav. Pharmacol.* 6 810–814. 10.1097/00008877-199512000-00006 11224384

[B82] Radke A. K. Sneddon E. A. Frasier R. M. Hopf F. W. (2021). Recent Perspectives on sex differences in compulsion-like and binge alcohol drinking. *Int. J. Mol. Sci.* 22:3788. 10.3390/ijms22073788 33917517 PMC8038761

[B83] Rehm J. Mathers C. Popova S. Thavorncharoensap M. Teerawattananon Y. Patra J. (2009). Global burden of disease and injury and economic cost attributable to alcohol use and alcohol-use disorders. *Lancet* 373 2223–2233. 10.1016/S0140-6736(09)60746-7 19560604

[B84] Rhodes J. S. Ford M. M. Yu C. H. Brown L. L. Finn D. A. Garland T. Jr. (2007). Mouse inbred strain differences in ethanol drinking to intoxication. *Genes Brain Behav.* 6 1–18. 10.1111/j.1601-183X.2006.00210.x 17233637

[B85] Robbins T. W. (2002). The 5-choice serial reaction time task: Behavioural pharmacology and functional neurochemistry. *Psychopharmacology (Berl)* 163 362–380. 10.1007/s00213-002-1154-7 12373437

[B86] Rolls E. T. (2016). Functions of the anterior insula in taste, autonomic, and related functions. *Brain Cogn.* 110 4–19. 10.1016/j.bandc.2015.07.002 26277487

[B87] Sanchez-Roige S. Baro V. Trick L. Pena-Oliver Y. Stephens D. N. Duka T. (2014a). Exaggerated waiting impulsivity associated with human binge drinking, and high alcohol consumption in mice. *Neuropsychopharmacology* 39 2919–2927. 10.1038/npp.2014.151 24947901 PMC4229569

[B88] Sanchez-Roige S. Pena-Oliver Y. Ripley T. L. Stephens D. N. (2014b). Repeated ethanol exposure during early and late adolescence: Double dissociation of effects on waiting and choice impulsivity. *Alcohol. Clin. Exp. Res.* 38 2579–2589. 10.1111/acer.12535 25346503

[B89] Saunders B. Farag N. Vincent A. S. Collins F. L. Jr. Sorocco K. H. Lovallo W. R. (2008). Impulsive errors on a Go-NoGo reaction time task: Disinhibitory traits in relation to a family history of alcoholism. *Alcohol. Clin. Exp. Res.* 32 888–894. 10.1111/j.1530-0277.2008.00648.x 18373725 PMC2836919

[B90] Schumann G. Coin L. J. Lourdusamy A. Charoen P. Berger K. H. Stacey D. (2011). Genome-wide association and genetic functional studies identify autism susceptibility candidate 2 gene (AUTS2) in the regulation of alcohol consumption. *Proc. Natl. Acad. Sci. U.S.A.* 108 7119–7124. 10.1073/pnas.1017288108 21471458 PMC3084048

[B91] Seemiller L. R. Gould T. J. (2020). The effects of adolescent alcohol exposure on learning and related neurobiology in humans and rodents. *Neurobiol. Learn. Mem.* 172:107234. 10.1016/j.nlm.2020.107234 32428585 PMC7797082

[B92] Seif T. Chang S. J. Simms J. A. Gibb S. L. Dadgar J. Chen B. T. (2013). Cortical activation of accumbens hyperpolarization-active NMDARs mediates aversion-resistant alcohol intake. *Nat. Neurosci.* 16 1094–1100. 10.1038/nn.3445 23817545 PMC3939030

[B93] Semenova S. (2012). Attention, impulsivity, and cognitive flexibility in adult male rats exposed to ethanol binge during adolescence as measured in the five-choice serial reaction time task: The effects of task and ethanol challenges. *Psychopharmacology (Berl)* 219 433–442. 10.1007/s00213-011-2458-2 21881872 PMC4018242

[B94] Smith M. L. Walcott A. T. Heinricher M. M. Ryabinin A. E. (2017). Anterior Cingulate cortex contributes to alcohol withdrawal- induced and socially transferred hyperalgesia. *eNeuro* 4:ENEURO.0087-17.2017. 10.1523/ENEURO.0087-17.2017 28785727 PMC5526654

[B95] Somkuwar S. S. Quach L. W. Quigley J. A. Purohit D. C. Fannon M. J. Koob G. F. (2018). Ethanol reinforcement elicits novel response inhibition behavior in a rat model of ethanol dependence. *Brain Sci.* 8:119. 10.3390/brainsci8070119 29949891 PMC6070985

[B96] Spiga S. Talani G. Mulas G. Licheri V. Fois G. R. Muggironi G. (2014). Hampered long-term depression and thin spine loss in the nucleus accumbens of ethanol-dependent rats. *Proc. Natl. Acad. Sci. U.S.A.* 111 E3745–E3754. 10.1073/pnas.1406768111 25122682 PMC4156750

[B97] Starski P. Hong S. I. Peyton L. Oliveros A. Wininger K. Hutchison C. (2020). Ethanol induces maladaptive impulse control and decreased seeking behaviors in mice. *Addict. Biol.* 25:e12754. 10.1111/adb.12754 31012186 PMC6810713

[B98] Starski P. Peyton L. Oliveros A. Heppelmann C. J. Dasari S. Choi D. S. (2019). Proteomic profile of a chronic binge ethanol exposure model. *J. Proteome Res.* 18 3492–3502. 10.1021/acs.jproteome.9b00394 31329447

[B99] Strickland J. C. Johnson M. W. (2021). Rejecting impulsivity as a psychological construct: A theoretical, empirical, and sociocultural argument. *Psychol. Rev.* 128 336–361. 10.1037/rev0000263 32969672 PMC8610097

[B100] Sutera F. M. De Caro V. Cannizzaro C. Giannola L. I. Lavanco G. Plescia F. (2016). Effects of DA-Phen, a dopamine-aminoacidic conjugate, on alcohol intake and forced abstinence. *Behav. Brain Res.* 310 109–118. 10.1016/j.bbr.2016.05.006 27155501

[B101] Tapert S. F. Schweinsburg A. D. Barlett V. C. Brown S. A. Frank L. R. Brown G. G. (2004). Blood oxygen level dependent response and spatial working memory in adolescents with alcohol use disorders. *Alcohol. Clin. Exp. Res.* 28 1577–1586. 10.1097/01.ALC.0000141812.81234.A6 15597092

[B102] Torregrossa M. M. Xie M. Taylor J. R. (2012). Chronic corticosterone exposure during adolescence reduces impulsive action but increases impulsive choice and sensitivity to yohimbine in male Sprague-Dawley rats. *Neuropsychopharmacology* 37 1656–1670. 10.1038/npp.2012.11 22334120 PMC3358734

[B103] Van Skike C. E. Diaz-Granados J. L. Matthews D. B. (2015). Chronic intermittent ethanol exposure produces persistent anxiety in adolescent and adult rats. *Alcohol. Clin. Exp. Res.* 39 262–271. 10.1111/acer.12617 25684048 PMC4331453

[B104] Vaughan C. L. Stangl B. L. Schwandt M. L. Corey K. M. Hendershot C. S. Ramchandani V. A. (2019). The relationship between impaired control, impulsivity, and alcohol self-administration in nondependent drinkers. *Exp. Clin. Psychopharmacol.* 27 236–246. 10.1037/pha0000247 30688502 PMC6776085

[B105] Wang S. Lai X. Deng Y. Song Y. (2020). Correlation between mouse age and human age in anti-tumor research: Significance and method establishment. *Life Sci.* 242:117242. 10.1016/j.lfs.2019.117242 31891723

[B106] Weafer J. de Wit H. (2014). Sex differences in impulsive action and impulsive choice. *Addict. Behav.* 39 1573–1579. 10.1016/j.addbeh.2013.10.033 24286704 PMC4012004

[B107] Weafer J. De Arcangelis J. de Wit H. (2015). Sex differences in behavioral impulsivity in at-risk and non-risk drinkers. *Front. Psychiatry* 6:72. 10.3389/fpsyt.2015.00072 26029121 PMC4429551

[B108] Wegner S. A. Pollard K. A. Kharazia V. Darevsky D. Perez L. Roychowdhury S. (2017). Limited excessive voluntary alcohol drinking leads to liver dysfunction in mice. *Alcohol. Clin. Exp. Res.* 41, 345–358. 10.1111/acer.13303 28103636 PMC5636002

[B109] White A. Castle I. J. Chen C. M. Shirley M. Roach D. Hingson R. (2015). Converging patterns of alcohol use and related outcomes among females and males in the United States, 2002 to 2012. *Alcohol. Clin. Exp. Res.* 39 1712–1726. 10.1111/acer.12815 26331879

[B110] White M. G. Panicker M. Mu C. Carter A. M. Roberts B. M. Dharmasri P. A. (2018). Anterior cingulate cortex input to the claustrum is required for top-down action control. *Cell Rep.* 22 84–95. 10.1016/j.celrep.2017.12.023 29298436 PMC5779631

[B111] Wilcox C. E. Dekonenko C. J. Mayer A. R. Bogenschutz M. P. Turner J. A. (2014). Cognitive control in alcohol use disorder: Deficits and clinical relevance. *Rev. Neurosci.* 25 1–24. 10.1515/revneuro-2013-0054 24361772 PMC4199648

[B112] Wilcox M. V. Cuzon Carlson V. C. Sherazee N. Sprow G. M. Bock R. Thiele T. E. (2014). Repeated binge-like ethanol drinking alters ethanol drinking patterns and depresses striatal GABAergic transmission. *Neuropsychopharmacology* 39 579–594. 10.1038/npp.2013.230 23995582 PMC3895236

[B113] Wilhelm C. J. Reeves J. M. Phillips T. J. Mitchell S. H. (2007). Mouse lines selected for alcohol consumption differ on certain measures of impulsivity. *Alcohol. Clin. Exp. Res.* 31 1839–1845. 10.1111/j.1530-0277.2007.00508.x 17850219

[B114] Winstanley C. A. (2011). The utility of rat models of impulsivity in developing pharmacotherapies for impulse control disorders. *Br. J. Pharmacol.* 164 1301–1321. 10.1111/j.1476-5381.2011.01323.x 21410459 PMC3229763

[B115] Winstanley C. A. Eagle D. M. Robbins T. W. (2006). Behavioral models of impulsivity in relation to ADHD: Translation between clinical and preclinical studies. *Clin. Psychol. Rev.* 26 379–395. 10.1016/j.cpr.2006.01.001 16504359 PMC1892795

[B116] Yankelevitz R. L. Mitchell S. H. Zhang Y. (2012). Gender differences in factors associated with alcohol drinking: Delay discounting and perception of others’ drinking. *Drug Alcohol Depend.* 123 273–276. 10.1016/j.drugalcdep.2011.11.012 22154359 PMC3925447

[B117] Zakiniaeiz Y. Scheinost D. Seo D. Sinha R. Constable R. T. (2017). Cingulate cortex functional connectivity predicts future relapse in alcohol dependent individuals. *Neuroimage Clin.* 13 181–187. 10.1016/j.nicl.2016.10.019 27981033 PMC5144743

